# A refined guide for aging muskoxen (*Ovibos moschatus*) based on mandibular examination

**DOI:** 10.1371/journal.pone.0328994

**Published:** 2025-09-24

**Authors:** Erica Suitor, Eleanor Dickinson, John Scheels, Mathieu Pruvot, Fabien Mavrot, Ekaluktutiak Hunters and Trappers Organization, Kugluktuk Angoniatit Association, Olokhaktomiut Hunters and Trappers Organization, Tracy Davison, Lisa-Marie Leclerc, Susan Kutz

**Affiliations:** 1 Faculty of Veterinary Medicine, University of Calgary, Calgary, Alberta, Canada; 2 Milwaukee County Zoo, Milwaukee, Wisconsin, United States of America; 3 Ekaluktutiak Hunters and Trappers Organization, Cambridge Bay, Nunavut, Canada; 4 Kugluktuk Angoniatit Association, Kugluktuk, Nunavut, Canada; 5 Olokhaktomiut Hunters and Trappers Organization, Ulukhaktok, Northwest Territories, Canada; 6 Department of Environment and Natural Resources, Government of the Northwest Territories, Inuvik, Northwest Territories, Canada; 7 Department of Environment, Government of Nunavut, Kugluktuk, Nunavut, Canada; Senckenberg Gesellschaft fur Naturforschung, GERMANY

## Abstract

Accurately aging wildlife is essential for effective management and conservation efforts because it supports the estimation of demographic parameters used to model population dynamics and determine harvest quotas. Currently, accurately aging muskoxen is limited by the lack of validated and standardized protocols specific to this species. We investigated three methods for aging muskoxen: tooth eruption pattern, mandible morphometrics, and cementum annuli analysis (CAA). We examined 260 mandibles from community-harvested muskoxen with known harvest dates from Nunavut and the Northwest Territories and radiographed 89 of these mandibles with erupting teeth to track eruption stages. From these data, we developed a key to estimate age of muskoxen from newborn until all permanent teeth have completely erupted (60 months). Next, we assessed the relationship between muskox mandible morphometrics and age using 178 archived mandibles from Banks Island, Northwest Territories. Caudal mandible length was the strongest predictor of age in months (Adjusted R^2^ = 0.917) up to 5 years old (60 months), after which growth was negligible. This regression model included linear, quadratic, and interaction terms for caudal mandible length with sex. To evaluate accuracy of CAA for aging, we compared cementum results from incisors of 14 captive muskoxen to their known age using linear regression (Adjusted R^2^ = 0.847). We applied this model, fitted to captive muskox data, to predict the age of 32 community harvested adult muskoxen over 60 months old (5 yo^+^) using cementum age results. While there was a tendency to underestimate age, this method provided a more informative estimate than classifying all animals as adults once all teeth have erupted. Integrating these methods, we developed a decision tree to guide aging based on the putative age class and available sample type. This framework improves age estimation accuracy for harvested muskoxen, supporting population models and enabling more effective management and conservation.

## Introduction

Individual animal age is a fundamental variable to understand population demographics and dynamics [1–3]. Effective management of wild ungulates draws on age structure data to estimate key demographic parameters such as reproduction, growth, and survival, which inform management strategies [3–5]. Consequently, reliable methods for aging live and dead animals are essential in many aspects of wildlife research and management [6].

Teeth are valuable tools for estimating the age of mammals because tooth development is conserved (i.e., primary teeth are replaced by permanent teeth in a predictable sequence) and teeth are resistant to decomposition [7,8]. Tooth development and mineralization follows a species-specific sequence which consists of thickening of the oral epithelium, formation of dental buds, the cap stage where teeth begin to develop, and the bell stage where the final tooth crown shape becomes apparent [9–11]. Tooth eruption and wear patterns are widely used for aging wildlife because teeth are often preserved in specimens, easy to access, and straightforward to evaluate [8,12–15]. Additionally, in species with a discrete birthing season, tooth eruption patterns can provide crucial information about the time or season of death, making them especially useful in post-mortem examinations where the date of death is unknown [16,17].

As teeth develop and erupt, the mandible bone also grows. Mandible morphometrics can supplement or serve as an alternative to tooth eruption patterns for age estimation in ungulates [18,19]. Mandibular morphometry has been used for age and sex determination of caribou (*Rangifer tarandus*) and has been briefly explored in muskoxen (*Ovibos moschatus*), demonstrating a gradual increase in total mandible length until fully grown [20–22].

Cementum annuli analysis (CAA) is a method widely used to age ungulates [2,12,23]. Cementum is a mineralized tissue deposited on tooth roots in distinct density patterns, forming a wider, translucent layer in summer and a narrow, darker, hypermineralized layer in winter [6]. A range of factors can influence cementum production, including mechanical stresses [24,25], disease and nutritional stress [26,27] and age [28,29]. Cementum annuli analysis involves counting the annually formed deposit lines, then adding the age at which the examined tooth erupted [30]. While CAA can be applied to younger individuals, tooth eruption provides a more accurate, cost-effective, and efficient method for aging in earlier life stages [31,32]. Cementum annuli analysis is a widely accepted aging technique for several ungulate species, including caribou [20], white-tailed deer (*Odocoileus virginianus)* [23], and moose (*Alces alces*) [33], but its validity and applicability has not been evaluated for muskoxen.

In the Canadian Arctic, muskoxen play a critical role in the ecosystem and local culture [34–36]. However, two of the previously largest muskox populations, on Banks and Victoria Islands in the Canadian Archipelago, have significantly declined in recent decades [37–40]. Accurately aging muskoxen would inform the interpretation of population trajectories and understanding cohort dynamics [41–43]. While the average life expectancy of wild muskoxen remains uncertain, estimates from community consultations in Greenland suggest that bulls live around 11 years, ranging from 9–13 years, and cows around 18 years, ranging from 15–20 years [38,44]. In captivity, muskoxen have lived beyond 20 years [45]. Previous studies have established aging methods for muskoxen up to 4 years old (yo) using annual age categories that primarily rely on tooth eruption patterns and horn growth [22,46] and provide limited information about the seasonal changes in tooth development and eruption. To age muskoxen beyond their growth period, only one formal report described the use of CAA, and the results were inconclusive due to the limited number of known-age animals [46].

The goal of this study was to refine protocols for aging muskoxen based on three methods of mandibular examination. Our first aim was to document tooth development and eruption patterns on a monthly or seasonal basis. Second, we sought to determine whether age could be estimated from mandible morphometrics. Our third aim was to assess the utility of aging adult muskoxen using CAA. Finally, we aimed to develop a practical integrated guide for aging muskoxen that would be accessible to community-based researchers as well as academic and government researchers.

## Materials and methods

Detailed protocols are available at protocols.io DOI: https://doi.org/10.17504/protocols.io.j8nlkdb6xg5r/v1

### Aging by tooth eruption pattern

#### Samples.

**Fig 1:**
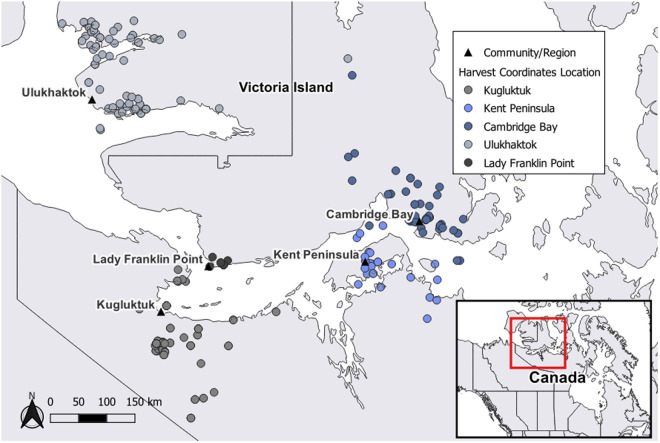
Study area and origin of samples. Geographic locations of muskox mandibles received from hunters in Nunavut and the Northwest Territories, Canada through a Community-based Wildlife Health Surveillance program (n = 260). Harvest community/region was unknown for three muskoxen (i.e., excluded from the map). Inset map depicts the region sampled in Canada. The map was created using country and province boundaries from naturalearthdata.com.

The first dataset included mandibles submitted by hunters through a Community-based Wildlife Health Surveillance (CBWHS) program. The CBWHS program was established to address concerns about declining muskox and caribou populations by incorporating multiple sources of knowledge [47]. The program operates in three Inuit communities: Kugluktuk and Ekaluktutiak (Cambridge Bay) in Nunavut (NU) and Ulukhaktok in the Northwest Territories (NT), Canada. Local hunters collect and submit samples, including mandibles, during subsistence and guided-outfitting hunts to assess various health indices for muskoxen and caribou [48]. We received 260 muskox mandibles collected from two distinct regions on the mainland and three regions on Victoria Island from 2014 to 2021 (Fig 1; S1 Table). Hunters recorded sex, date of death, harvest coordinates, and estimated age class. Each mandible had an assigned CBWHS ID and were frozen and stored at −20°C until further analyses. The map in Fig 1 was created with QGIS [49] using publicly available shapefiles from naturalearthdata.com.

Samples were obtained under the Wildlife Research Permits #2013–035, 2014–053, 2015–068, 2016–058, 2019–051, and 2020–045 for Nunavut; Wildlife Research Permits #WL500098, WL500158, WL500257, and WL500877 for the Northwest Territories; and the University of Calgary Veterinary Sciences Animal Care Committee Permits AC13–0121, AC18–0093, and AC22–0060.

#### Tooth eruption pattern by gross examination.

Mandibles were thawed and trimmed of excess tissue, including skin and muscle, and assigned an initial age class based on tooth eruption patterns using a dental aging key which identifies tooth eruption up to 4 yo (S2 Fig), adapted from Henrichsen and Grue [46]. In muskoxen, the mandible typically has eight primary incisors and six primary molars that complete eruption shortly after birth. During gross examination of incisors, premolars, and molars the eruption stage of each tooth was recorded as primary, erupting permanent, or permanent. Primary molars are the first set of molars that erupt as part of the primary dentition. They are later replaced by permanent premolars. Given that muskoxen are typically born between late April and early June, a standardized birth date of May 1^st^ was assigned for all individuals [46]. Following examination each mandible was photographed for documentation and subsequent reference.

#### Medical imaging to refine tooth development and eruption patterns.

**Fig 2:**
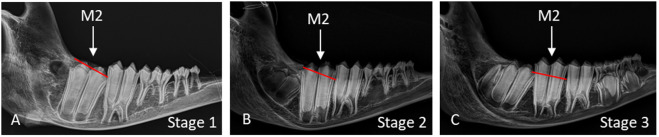
Stages of tooth eruption. Lateral radiographs of muskox mandibles illustrating the progression of molar 2 (M_2_) eruption, with the bone line indicated in red: (A) Eruption Stage 1–starting to erupt through the bone; (B) Eruption Stage 2—partially erupted through the bone; (C) Eruption Stage 3—complete eruption through both bone into oral cavity.

To explore the stages of tooth development, a subset of mandibles (n = 43) from young muskoxen, initially classified by age up to 4 yo, were scanned using computerized tomography (CT; GE Revolution Discovery CT scanner, GE Healthcare, USA) with 64 detectors (in Z direction at 0.625 mm), 0.625 mm section thickness, and tube settings of 120kV and 200mA. Results from the CT scans prompted further analysis of tooth eruption stages using radiographs and a larger sample size. An additional subset of mandibles (n = 89), with teeth still erupting, were radiographed using the InnoVet DXR digital X-ray system (Summit Industries, USA). To prevent interference with lateral radiographs of the left tooth row (i.e., cheek teeth [50]), the right side of each mandible was cut at the diastema. Lateral radiographs were then captured of the labial aspect of the left tooth row (except when not available) and dorsoventral radiographs of the incisors. These radiographs, along with accompanying photographs, were evaluated to assess the completion of tooth mineralization and the progression of eruption. Progression of eruption was simply classified as stage 1, 2, or 3 (Fig 2). Complete eruption (stage 3) was defined as the tooth having fully erupted through the bone and reaching its functional position within the oral cavity.

Age in months was estimated using tooth eruption patterns, along with the birth date, and date of death recorded on the CBWHS form. Mandibles were arranged chronologically from youngest to oldest and grouped by season of death—spring (April–June), summer (July–September), autumn (October–December), and winter (January–March)—to account for variability in tooth eruption timing due to unknown exact birth dates. Eruption through the bone was assessed for each tooth, with particular emphasis on the third molar (M_3_), as it is the last molar to erupt. Age estimates from these images were reviewed by a wildlife dentist (J. Scheels).

### Aging by mandible morphometrics

#### Samples.

The second dataset was used to assess the utility of mandible measurements for aging muskoxen. We examined 178 archived hemimandibles with known sex from Banks Island, NT (Table 1). These were collected between 1986 and 1992 during commercial harvests organized by the Sachs Harbour Hunters and Trappers Committee and Inuvialuit Development Corporation [51]. Along with other biological samples, the left mandibles were collected by Government of the Northwest Territories biologists. Mandibles were boiled to remove excess tissue and stored dry at ambient temperature. Samples were labelled with animal ID, sex, and date of death. The uniform cleaned and dried state of these mandibles allowed reliable morphometric measurements from a single muskox population. In contrast, the CBWHS mandibles were not used because the specimens were not consistently cleaned and preserved.

**Table 1:** Mandibles collected between 1986 to 1992 from Banks Island, Nunavut. Listed by sex and confirmed estimated age according to tooth eruption pattern.

Age	Female	Male	Total
*Calf (1–12 mo)*	13	7	20
*1 yo (13–24 mo)*	7	19	26
*2 yo (25–36 mo)*	13	24	37
*3 yo (37–48 mo)*	22	21	43
*4 yo (49–60 mo)*	19	14	33
*5 yo^+^* *(> 60 mo)*	14	5	19
**Total**	**88**	**90**	**178**

**Fig 3:**
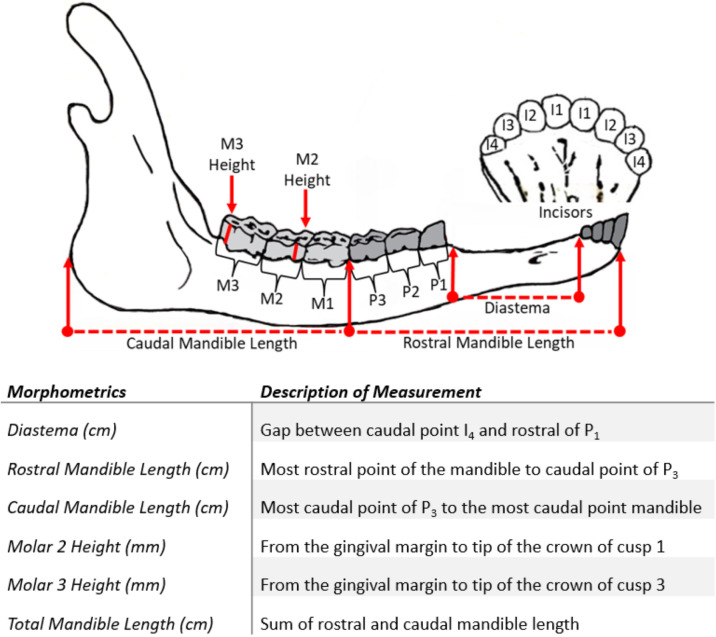
Morphometrics recorded for muskox mandibles and a description of each measurement. Adapted from a CircumArctic Rangifer Monitoring and Assessment (CARMA) protocol [52]. I = Incisor, P = Premolar, M = Molar.

First, each mandible was aged based on tooth eruption patterns. The diastema, rostral, and caudal mandible lengths were measured with calipers (Fig 3). Rostral and caudal mandible lengths were summed to calculate the total mandible length. A ruler was used to measure height of molar 2 (M_2_) and M_3_, from the cervical junction between the root and crown to the crown tip.

### Statistical analysis: mandible morphometrics

The linearity of relationships between predictors and age was assessed using scatter plots and residual plots. Collinearity among predictors was evaluated using the variance inflation factor (VIF); predictors with a VIF greater than 5, indicating collinearity [53], were excluded to simplify the model. When collinearity was detected, independent models were run to identify the best predictors of age. Molar heights (M_2_ and M_3_) and other variables not directly related to mandible morphometrics were excluded from analysis due to unavailability of measurements before eruption and the variability introduced by tooth wear patterns [15,54].

Model selection was performed using the Akaike Information Criterion (AIC) to identify the best-fitting models. Linear, quadratic, and interaction terms with sex were evaluated for each predictor. Quadratic terms were included to address non-linear patterns observed during initial visual assessments. Interaction terms with sex were tested to determine whether the relationships between predictors and age differed by sex. The accuracy of the final model was evaluated using mean absolute error (MAE), which measures average prediction error, and root mean squared error (RMSE), which gives greater weight to larger errors. Residual diagnostics, including Q-Q plots and residuals versus fitted value plots, were examined to assess normality, homoscedasticity. All analyses were conducted in R version 4.3.2 [55].

### Aging by cementum annuli analysis

#### Samples.

To investigate the accuracy and broader applicability of CAA for aging after all teeth have erupted, we used a third dataset that incorporated known-age captive muskoxen alongside a subset of wild muskoxen from the CBWHS program. Incisors were extracted from the mandibles of 21 dead captive muskoxen (five females, 10 males, six of unknown sex) of known age. Muskoxen originated from five facilities: Muskox Farm in Palmer, Alaska, USA; the University of Alaska Fairbanks Large Animal Research Station (UAF-LARS), Fairbanks, Alaska, USA; Assiniboine Park Zoo in Winnipeg, Manitoba, Canada; Western College of Veterinary Medicine in Saskatoon, Saskatchewan, Canada; University of Calgary Faculty of Veterinary Medicine in Calgary, Alberta, Canada. If available, both central incisors (I_1_) were extracted from each mandible.

We selected 32 adult CBWHS muskoxen of unknown age (5 yo^+^) for CAA based on the degree of tooth wear to capture a diverse range of potential ages. These individuals were categorized into broad age classes—young adult, prime adult, or senescent adult—using criteria such as dental staining, incisor and lingual crest wear on molars, and signs of gumline inflammation or staining (Table 2). Shorter tooth height and increased staining were used as indicators of advancing age [15].

**Table 2:** Age categories assigned to adult muskoxen.

Age Category	Incisor Wear	Molar Wear
*Young Adult*	Minimal to no incisor wear; little to no staining	Pointed lingual crests and well–defined lines of dentine
*Prime Adult*	Moderate incisor wear with some staining	Moderate wear on M_1_ and M_2_ crests; slight wear beginning on M_3_
*Senescent Adult*	Heavy incisor wear with staining	Heavy wear on crests of all molars

Age category was assigned to muskox mandibles from adults of unknown age collected from the Community-based Wildlife Health Surveillance program. Each animal’s age category was determined based on tooth wear and staining (adapted from Hinton et al. [15]).

Following Matson’s Laboratory (Manhattan, MT, USA) recommended methods to extract incisors [24], two lateral cuts were made through the gingiva adjacent to each I_1_, ensuring not to damage the root. A dental probe was used to loosen the attachment of the periodontal ligament, and then dental pliers were applied to extract the incisor. Teeth were placed in paper envelopes labeled with the animal’s ID number, sex, harvest location, and death date then stored at room temperature to dry any soft tissue. Once dried, the left I_1_ (or, if unavailable, another incisor) was placed in a new envelope with a new ID number to ensure blinding of animal information once sent to Matson’s Laboratory.

#### Cementum annuli analysis.

A total of 53 incisors (I_1_) were submitted to Matson’s Laboratory, Manhattan, MT, USA, for age estimation of adult muskoxen using CAA. This included 21 incisors from captive muskoxen (Left I_1_, n = 13; Right I_1_, n = 7; Unknown I_1_, n = 1), 32 incisors from adult CBWHS muskoxen (Left I_1_, n = 27; Right I_1_, n = 5).

The incisors were decalcified in hydrochloric acid, fixed in formalin, embedded using the paraffin method [56], and sectioned at 14μm using a microtome (Model RM 2155; Leica Biosystems, Buffalo Grove, IL, USA). Each incisor was sectioned twice to provide two age estimates per individual, similar to Lundervold et al. [57]. These sections were mounted on slides, Giemsa stained (RICCA Chemical Co., Arlington, TX, USA), and cover slipped for microscopic examination of cementum annuli. The two sections were examined, using a compound microscope at x4, x10, or x20 magnification, one week apart by an experienced technician (S. Hannebaum) who was blinded to the sample ID. Age estimates were made in accordance with the Matson’s Laboratory North American muskox I_1_ model where the first defined annulus separated from the dentine is interpreted as year 3 and subsequent annuli are counted as observed. A birth date of May 1^st^ was assumed, and the interpretation of the final cementum annuli was based on season or date of death (Matson’s Laboratory, unpublished report). A 27 yo female was excluded from the analysis due to her uncommon old age. Six incisors from the Saskatchewan herd had poor cementum quality resulting in inconclusive age estimates. These data were excluded, leaving a total of 14 CAA results from captive muskoxen of known age for further analysis.

#### Statistical analysis: Cementum annuli analysis.

To check for consistency of age estimate within teeth, Pearson’s correlation coefficient was calculated between the estimated age of the duplicate teeth sections. Following this, average CAA–estimated ages of captive muskoxen were compared to their known age using a linear regression model to determine the accuracy. We also examined the deviation from the expected relationship (actual age equals cementum age). The accuracy of the model was assessed with MAE and RMSE. Finally, this linear regression model was applied to the adult CBWHS samples selected for CAA (n = 32) to predict age based on cementum age. To quantify the uncertainty in age estimation, we included the 95% confidence intervals, which represent the range of values likely to encompass the actual age.

## Results

### Aging by tooth eruption pattern

**Fig 4:**
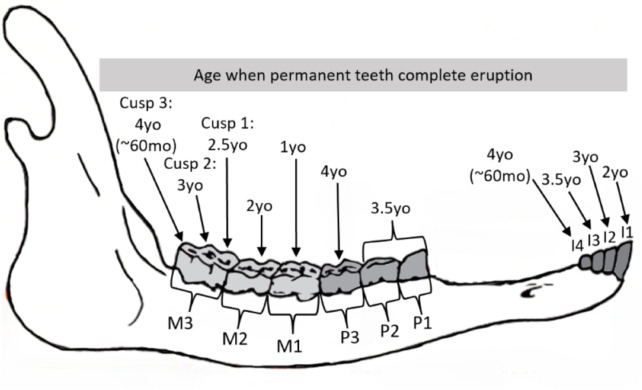
Diagram of a muskox mandible and age when each permanent tooth has completely erupted. Mandible outline adapted from a CircumArctic Rangifer Monitoring and Assessment (CARMA) protocol [52]. Abbreviations yo = years old, mo = months old, I = Incisor, P = Premolar, M = Molar.

**Fig 5:**
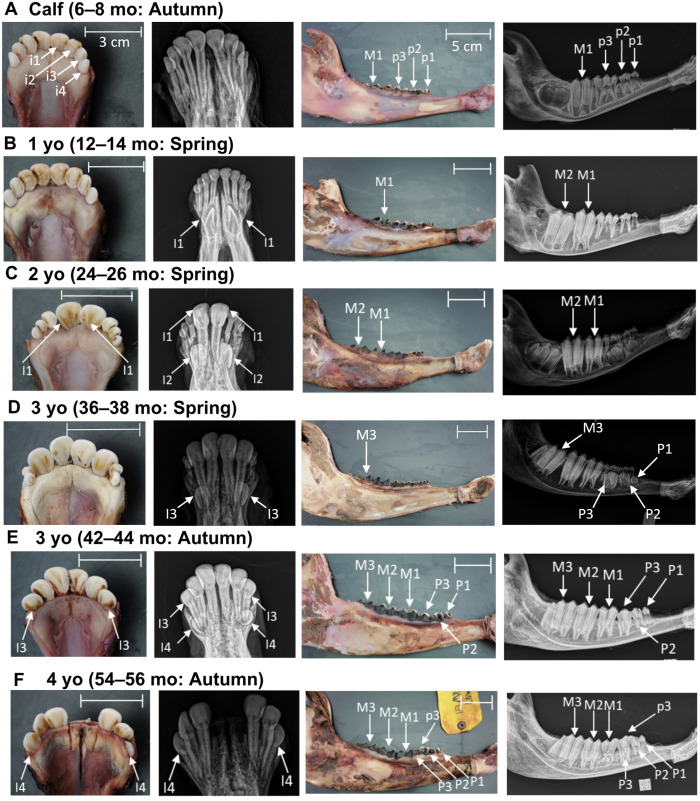
Timeline of tooth eruption shown in radiographs and photographs of muskox incisor arcades and mandibles of selected ages for each year of tooth (A–F). Primary incisors and molars are denoted with a lowercase “i” and “p” and permanent incisors and premolars are denoted with capital “I” and “P”, respectively. Permanent molars are labeled with a capital “M”. Eruption stage 1–starting to erupt through the bone; stage 2—partially erupted through the bone; stage 3—complete eruption through both bone into oral cavity. The scale bar for the incisor arcade measures 3 cm and the scale bar for the lateral view of the molars measures 5 cm. The age is provided in years old (yo) and months old (mo). The season beside each age when the animal was harvested. Missing incisors are either pre-mortem fractures or were extracted for cementum annuli analysis.

Age was estimated for 260 mandibles collected through the CBWHS using tooth eruption patterns: 26 aged as a calf (1–12 mo), 32 aged as 1 yo (13–24 mo), 19 aged as 2 yo (25–36 mo), 13 aged as 3 yo (37–48 mo), 20 aged as 4 yo (49–60 mo), and 150 aged as 5 yo^+^ (> 60 mo). Age when each permanent tooth completes eruption was summarized (Fig 4) and eruption for a specific age for each year was shown visually (Fig 5). Individuals were aged from calves (1–2 months) up to 5 years (57–60 months), as detailed in S3 Table. Beyond this point, all permanent teeth had fully erupted, and individuals were classified as 5 yo^+^. The eruption stage of each tooth was summarized by age, in both months and years, through gross examination (S4 and S5 Table). Focusing on key eruption periods by tooth type, premolars (P_1_–P_3_) erupted between 33–35 months and 54–56 months. Radiographs provided clear visualization of premolar eruption stages, which are often obscured by primary precursors during gross examination. Molar eruption spanned a longer period, beginning with M_1_ at 3–5 months and M_3_ completing eruption by 57–60 months. Specifically, the third cusp of M_3_ began erupting through the bone between 42–44 months (Fig 5) and completed eruption by 57–60 months.

**Fig 6:**
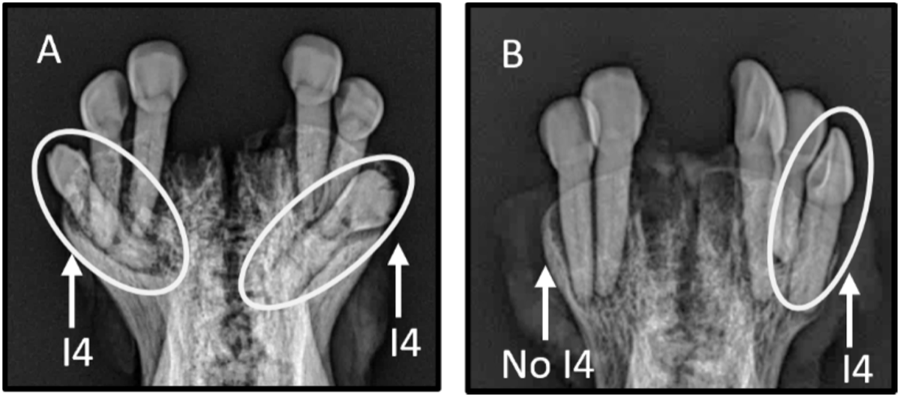
Radiographic images of muskox incisor arcades from Ulukhaktok, NU. These were adult muskoxen (5 years or older) based on molar eruption and wear. (A) The permanent I_4_ are fully developed but have not erupted. (B) The left I_4_ has not developed while the right I_4_ has erupted. The first incisors (I_1_) were extracted for cementum annuli analysis before radiographing.

Permanent incisors 1 to 3 erupted between 21–23 months and 42–44 months. Permanent incisor 4 (I_4_) erupted ranging between 48–60 months however, it did not always erupt, and this differed by geographic location. The fourth incisor had not erupted in 44.6% (33/74) of muskoxen aged 5 yo^+^ on Victoria Island, compared to 2.74% (2/73) of mainland muskoxen from Kugluktuk and Kent Peninsula. When compared in radiographs, I_4_ was developed but not erupted in 32.7% (16/49) and not developed in 18.4% (9/49) of radiographed muskoxen aged 5 years or older from Victoria Island (Fig 6).

### Aging by mandible morphometrics

The variance inflation factor (VIF) exceeded five for diastema, rostral mandible length, caudal mandible length, and total mandible length. The relationship between age in months and muskox mandible morphometrics was analyzed using linear, quadratic, interaction (sex), and full models for four morphometric variables: diastema, rostral mandible length, caudal mandible length, and total mandible length (a total of 16 models). Models were ranked using AIC to identify the best-fit model.

**Fig 7:**
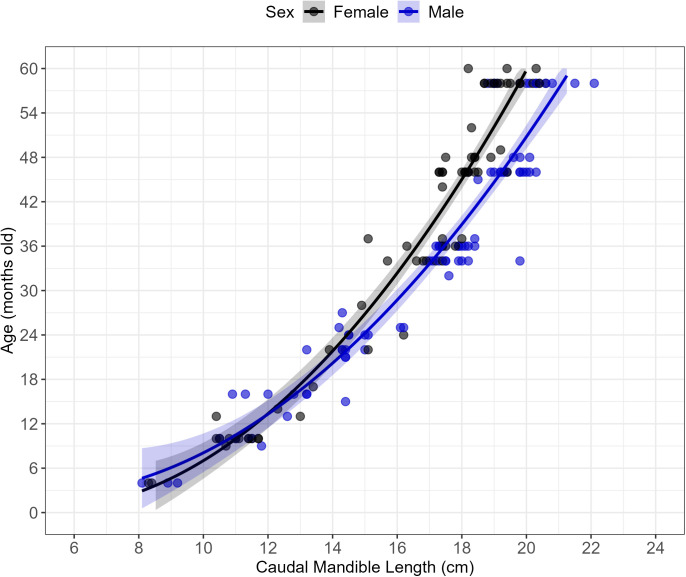
The relationship between age in months and caudal mandible length for wild muskoxen. Mandibles were collected from 88 females and 90 males harvested on Banks Island from 1986 to 1992. Age was determined from tooth eruption patterns, and its relationship with caudal mandible length was modeled using a regression that included linear and quadratic terms for caudal mandible length (cm), sex, and their interactions. Fitted regression lines (black for females and blue for males) and their 95% confidence intervals (shaded regions) are shown, with predictions and intervals truncated to a maximum age of 60 months. Observed ages from tooth eruption patterns are represented by individual data points.

The lowest AIC was observed for the full model using caudal mandible length (AIC = 930), indicating it provided the strongest fit among all tested models. This model included linear, quadratic, and interaction terms for caudal mandible length with sex (Fig 7). The model explained a substantial proportion of the variation in age (Adjusted R^2^ = 0.917, F = 423 (4,149)). The mean absolute error (MAE) between predicted and actual ages for caudal mandible length was 3.57 months, and the root mean squared error (RMSE) was 4.75 months. The quadratic–only model using caudal mandible length (AIC = 945) and the full model using total mandible length (AIC = 960) were the next best, though their performance was weaker. All other morphometric variables performed poorly (ΔAIC > 30) compared to the best model.

### Aging by cementum annuli analysis

Age was estimated for 15 captive muskoxen using CAA (S6 Table). We used the average age from both tooth sections for 14 individuals in the linear regression. There was a significant positive relationship between the actual age of captive muskoxen and their cementum–derived age estimate (Adjusted R² = 0.847, F = 73.2, p < 0.0001; Fig 7A). The MAE between predicted and actual ages was 2.75 years, while the RMSE was 3.40 years. Age estimates ranged from 4.5 to 16 years and consistently underestimated the actual age by 1 to 8 years (Fig 7A).

**Fig 8:**
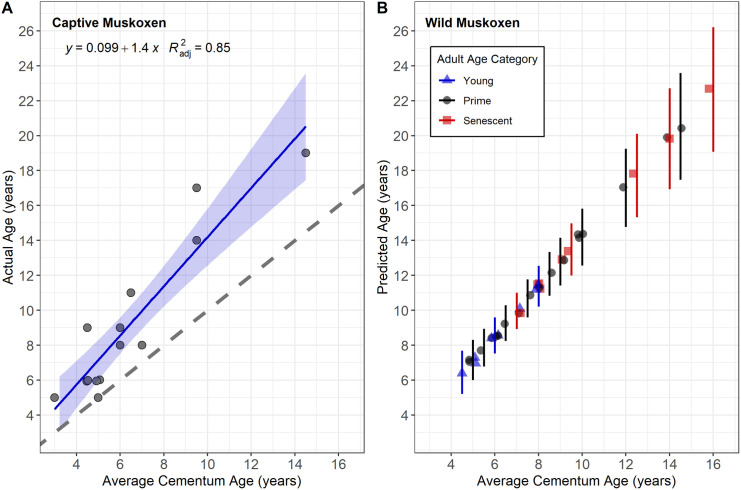
The relationship between cementum age and actual age or predicted age of muskoxen. (A) Data from incisors of 14 captive muskoxen with known ages. Cementum age was the average between two sections of the same tooth. The regression equation and adjusted R^2^ value are displayed on the plot. The blue regression line indicates the trend, and the grey shaded area represents the 95% confidence interval. The dashed line indicates a perfect linear relationship with no deviation. (B) Data from 32 adult (5 yo^+^) muskoxen harvested through the Community-based Wildlife Health Surveillance (CBWHS) program in Nunavut and the Northwest Territories. Predicted values were derived using the average cementum age and the fitted linear model from panel A. Age categories were assigned based on tooth wear before cementum annuli analysis. Vertical bars indicate 95% confidence interval.

For the wild adult CBWHS muskoxen (n = 32), age was predicted using the linear model fitted on data from captive muskoxen (Fig 8A) and ranged from 6.45 to 22.6 years (Fig 8B). The mean predicted ages were 8.82 years for young adults (95% CI = 7.70 to 9.95), 11.9 years for prime adults (95% CI = 10.4 to 13.4), and 14.5 years for senescent adult (95% CI = 12.7 to 16.3).

### Integrated aging guide

**Fig 9:**
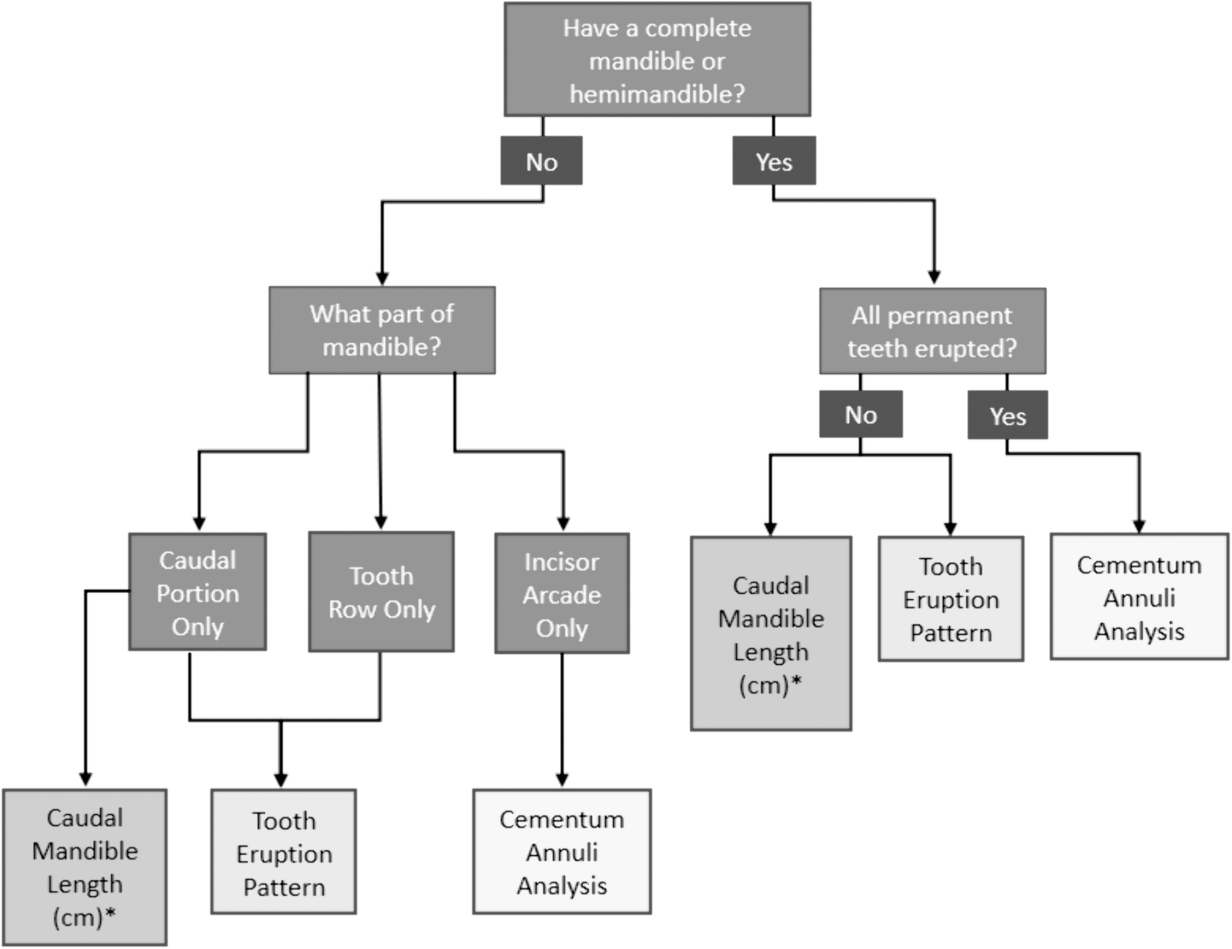
Decision tree for selecting appropriate aging method for muskoxen based on available sample. *Sex is required to estimate age since both total and caudal length differ for muskoxen 3 years and older.

Combining all three aging methods, we developed a decision tree to guide selection of the most appropriate method for aging depending on the type of sample available (Fig 9). If a full mandible or hemimandible is available, tooth eruption patterns can age muskoxen accurately from newborn up to 5 years. Caudal mandible length can provide age estimates in months up to 5 yo if the sex of the animal is known. To estimate age of muskoxen 5 years and older, CAA of I_1_ is recommended.

## Discussion

We have described and evaluated three methods for aging muskoxen using mandibles: tooth eruption pattern, mandible morphometrics, and CAA. Each method has unique strengths and limitations, making them suitable for different age ranges and specific contexts, thereby complementing one another to improve overall accuracy in age estimation.

### Aging by tooth eruption pattern

Tooth eruption patterns are commonly used to age ungulates due to predictable development, ease of examination, and correlation with growth [8,12,14]. In our study, we found molars the most consistent indicators of age in muskoxen, providing age estimation up to and including 4 years when the tooth row is available. In contrast, documenting incisor eruption was complicated by limited data during important periods of growth, as muskoxen were rarely harvested during the summer months. In other ruminants like red deer (*Cervus elaphus)* and roe deer (*Capreolus capreolus),* however, incisor eruption occurs more rapidly and variably than molar eruption [58,59], thus perhaps limiting its value for finer scale (i.e., month/season) aging. While our tooth eruption guide outlines the typical sequence in muskoxen, variations in timing can occur due to factors such as location, nutrition, environmental conditions, and sex [60–62]. Grouping animals into 3–month increments allowed us to account for some of this natural variability in eruption timing and birth dates.

We supplemented classical direct visual examination of tooth eruption with CT and radiographic imaging, allowing us to more precisely define tooth development and eruption stages that could not be tracked by gross examination alone. This approach was especially beneficial during the final stages of M_3_ eruption, where eruption stages were more apparent on radiographs than through visual inspection. This allowed us to classify individuals up to 60 months old, thus changing the previous 4 yo^+^ category to now a 5 yo^+^ category. The additional year that muskoxen can be aged provides more information on population age structure and for the exploration of cohort effects, which may be influenced by environmental factors during the year of birth [63]. Studies in fallow deer (*Dama dama),* red deer, and water deer (*Hydropotes inermi*) [64–67] have similarly used radiographs to more precisely define development and pre- and post-eruption stages.

Although the use of radiography required specialized equipment, it did provide additional insights. For example, it allowed us to differentiate between animals with an unerupted versus absent I_4_. Gross examination alone often fails to distinguish between types of hypodontia, such as a tooth that has developed but not yet erupted versus one that did not develop [68]. Additionally, while not applied in this study, radiographs did reveal narrowing and closure of root apices in older animals. Further examination of these patterns in the future may be valuable to further evaluate age in older individuals.

The eruption of I_4_ was inconsistent among the populations we examined. On Victoria Island, muskoxen aged 5 yo^+^ often had at least one unerupted or absent I_4_. This observation is consistent with reports of missing I_4_ in Greenland muskoxen [47]. In contrast, I_4_ eruption occurred more consistently in muskoxen from the mainland and this complemented molar eruption patterns for age estimation. In sheep, the absence of I_4_ is common with up to 15.4% of sheep reported to be lacking one or both I_4_ [69]. The variability in I_4_ development and eruption on Victoria Island may be influenced by a variety of factors, including small mandible size. Muskoxen on Victoria Island have a smaller incisor arcade width: total mandible length ratio compared to mainland populations (S7 Table), which may contribute to delayed or absent I_4_ development and eruption. Furthermore, these island muskoxen have exceptionally low genetic diversity—lower than their mainland counterparts [70–72]—which may play a role in developmental abnormalities [73,74].

The stability of teeth makes them a reliable record of an animal’s pre-mortem state and in animals where teeth are still erupting these may be able to inform on not just age, but also season of death [16,17]. When muskoxen die in the wild, while many bones are dispersed by predators and broken, the mandible is often found intact. This makes teeth valuable for postmortem applications, offering forensic and epidemiological insights into muskox mortalities (Box 1).

Box 1 **Using Muskox Aging Methods Based on Tooth Eruption Patterns for Forensic Investigations**

In June/July 2015, several muskoxen in varying stages of decomposition were found near Cambridge Bay, NU. The times of death for these animals were unknown. Three of 9 animals examined did not have fully erupted permanent teeth. This allowed us to estimate their ages and seasons of death using tooth eruption patterns and our age-by-month guide.

One muskox was estimated to be 21–23 months old based on molar 2 nearing stage 3 eruption. Assuming a birth date of May 1, the animal likely died in during winter (Jan–Mar). This seasonal timing ruled out algal bloom toxicity, a summer phenomenon, as a potential cause of death.

Accurately determining age and season of death is critical for wildlife forensic investigations. It provides valuable insights into muskox biology and disease ecology.

### Aging by mandible morphometrics

The replacement of primary teeth with permanent teeth cannot occur until the mandible reaches a critical size [75–77]. As a result, mandible morphometrics offer an alternative or complementary approach to tooth eruption patterns for aging muskoxen. Caudal mandible length was the strongest predictor of age during the mandible growth period (1–60 months). In instances where dental anomalies (i.e., missing or broken teeth) or atypical patterns occur, reliance on tooth eruption alone may lead to inaccuracies. By integrating mandible morphometrics with tooth eruption patterns, these limitations can be mitigated, as cross-validation enhances precision, reduces misclassification risk, and refines age estimates.

Muskoxen are sexually dimorphic, with adult females being 40–50% smaller than prime–aged males [78,79]. Differences in caudal and total mandible lengths between sexes become apparent around 3 years of age, with males showing significantly larger measurements. This highlights the importance of considering the animal’s sex for accurate age estimation beyond this stage. While our data demonstrate the utility of this method within a single population, variation in body size—and thus mandible size—may occur across populations due to factors such as geographic location, environmental conditions, and subspecies differences. While we would not expect different trends in the relationship between mandible morphometrics and age, it is crucial to validate these methods independently for each population to ensure accuracy and reliability.

### Aging by cementum annuli analysis

Our results, based on known aged captive animals, illustrated that CAA consistently underestimated the actual age of muskoxen and error increased with age. Few studies have statistically analyzed CAA error using known–age animals. Hamlin et al. [32] found no age–related errors in elk (*Cervus canadensis),* or mule deer (*Odocoileus hemionus)*. Veiberg et al. [6] reported that cementum age estimates in four northern cervid species were within ±1 year of the known age for 95% of individuals, although there was a tendency to underestimate age as animals grew older. As animals age, the narrowing space between cementum annuli can lead to underestimations, because older growth layers become less distinct [80]. Cementum production can also increase in response to mechanical strain, ensuring tooth attachment to the alveolar bone [25,26,81]. The variability of CAA, particularly in older animals, highlights the need for cautious interpretation. Muskoxen, which experience pronounced seasonality in the Arctic—with short, nutrient-rich summers and prolonged, nutritionally stressful winters—may produce faint or indistinct annuli due to their brief growth period. This could obscure the boundary between winter rest lines and summer growth layers. Furthermore, Rolandsen et al. [82] also noted that accuracy and repeatability varied between technicians, with less experienced technicians showing greater error, especially in aging older animals. Although our analysis relied on a single highly experienced technician, technical expertise remains an important consideration for future studies.

We used the relationship between the known age of captive muskoxen and CAA estimates to predict the age of wild muskoxen which we had previously classified into three age classes. While the predicted ages generally aligned with the assigned age classes, the greatest variability in CAA estimates was for prime–aged individuals, with overlap observed between young adults and senescent adults. Tooth wear, known to be inconsistent and highly variable among individuals and across locations (S8 Fig), may have contributed to this variability [83–85]. Notably, CAA has been shown to be more accurate than the commonly used tooth wear method [32,54].

### Limitations

Through an ongoing community-based health surveillance program of muskoxen, we were fortunate to have access to a relatively large sample size of mandibles. Such sample sizes are rarely available for wildlife. These mandibles were used for both examining eruption patterns and CAA. However, this sampling method introduces biases, particularly concerning the timing of harvests and the condition of the animals. Many samples were collected during autumn and winter, limiting our ability to fully assess seasonal variations in both tooth eruption and growth. More evenly distributed sampling across all seasons would be necessary to clarify the timing of incisor eruption. A limitation of our evaluation of CAA is its reliance on known–aged captive muskoxen; however, these individuals were sourced from broad geographic locations and husbandry practices and thus may represent a reasonable range of conditions.

### Integrated guide

Our goal was to refine aging methods for muskoxen using mandibular examination that would provide accurate methods for both hunters and biologists to consistently age animals. We designed an aging guide for muskoxen using three distinct techniques that use whole or partial mandibles, and our refined guide provides multiple tools for age estimation. Tooth eruption was the best tool for aging muskoxen up to 5 years of age because of its simplicity and consistency. When tooth eruption cannot be assessed, such as in cases of missing molars, mandible morphometrics are a useful alternative for this age range. While CAA can be used on young animals with primary teeth, we recommend using it only once all teeth have fully erupted with consideration of the underestimation of actual age. Prior to this stage, tooth eruption patterns are the preferred aging method as this method is precise and requires no sample preparation [33]. Finally, we produced a decision tree that can guide users to the most appropriate muskox aging method based on sample type available. Our integrated methods and detailed guide enhance the accuracy and practicality of muskox aging techniques, fill gaps in previous research, and offers a foundation for future studies and conservation efforts for this species.

## Supporting information

S1 Table Summary of mandibles from the Community-based Wildlife Health Surveillance program. Mandibles were collected between 2014 and 2021 listed by sex and harvest location (closest community or geographic landmark).
(PDF)

S2 Fig Original guide used to age muskoxen from tooth eruption. Adapted from Henrichsen and Grue (1980).
(TIF)

S3 Table Complete timeline of permanent tooth eruption. Radiographs and photographs of muskox incisor arcades and molars, showing known months of death and estimated age in years and months until all permanent teeth complete eruption. Primary incisors and molars were denoted with a lowercase “i” and “p” and permanent incisors and premolars were denoted with capital “I” and “P” respectively. Permanent molars are labeled with a capital “M”. Eruption stage 1–starting to erupt through the bone; stage 2—continuing to erupt through the bone; stage 3—complete eruption through both bone into oral cavity. Incisors that are missing either suffered pre-mortem fractures or were extracted for cementum annuli analysis.
(PDF)

S4 Table Tooth eruption patterns by age in months from gross examination. Stages of tooth eruption were assessed for each tooth, based on visual inspection of the mandibles. The stages are defined as follows: NE (No Eruption–white): tooth not erupted into the oral cavity; IE (Initial Eruption–lightest grey): first appearance of the tooth through the gingiva; PE (Partial Eruption–light grey): half of the tooth visible in the oral cavity; NCE (Near Complete Eruption–medium grey): tooth almost fully erupted through the gingiva; CE (Complete Eruption–dark grey): tooth completely emerged in its functional position in oral cavity. Primary incisors and molars were denoted with a lowercase “i” and “p” and permanent incisors and premolars were denoted with capital “I” and “P” respectively. Permanent molars are labeled with a capital “M”.
(PDF)

S5 Table Tooth eruption patterns for muskoxen across ages: calf, 1 yo, 2 yo, 3 yo, 4 yo, and 5 yo^+^. The stages are defined as follows: NE (No Eruption–white): Tooth not yet erupted into the oral cavity; PE (Partial Eruption–light grey): Less than half of the tooth visible in the oral cavity; NCE (Near Complete Eruption–medium grey): Tooth nearly emerged through the gingiva; CE (Complete Eruption–dark grey): Tooth completely emerged and in its functional position. Primary incisors and molars were denoted with a lowercase “i” and “p” and permanent incisors and premolars were denoted with capital “I” and “P” respectively. Permanent molars are labeled with a capital “M”.
(PDF)

S6 Table Cementum annuli analysis results for captive muskoxen. Estimates for two sections from the left I_1_ (if available) of muskoxen of known age from captive facilities. The grade indicates the confidence in the age estimate, where A–high confidence, B–some uncertainty, C–more uncertainty. Notes include information on abnormal histology, cementum damage.
(PDF)

S7 Table Difference in mandible morphometrics between locations. Ratio between mean incisor arcade width and total mandible length for adult muskoxen (5 years or older) from Victoria Island and the adjacent mainland. T-tests were done to compare ratio by sex.
(PDF)

S8 Fig Difference in cementum age estimates by age category and location. Estimated ages of 32 muskoxen from the Community-based Wildlife Health Surveillance program determined through cementum annuli analysis, shown by harvest community and assigned age category. Age categories were assigned based on visual assessment of tooth wear, with greater wear indicating older age classifications.
(TIF)

S9 Data Excel data file for Banks Island muskox mandible information. (XLSX)

S10 Data Excel data file for cementum annuli analysis from captive and wild muskoxen. (XLSX)
