# Supplementary material for: A refined guide for aging muskoxen (Ovibos moschatus) based on mandibular examination
Source: PLoS One. 2025 Sep 24;20(9):e0328994. doi: 10.1371/journal.pone.0328994 (PMC12459791; doi:10.1371/journal.pone.0328994)
Supplement: S1 Table — Mandibles were collected between 2014 and 2021 listed by sex and harvest location (closest community or geographic landmark). (PDF) [file pone.0328994.s001.pdf]

S1 Table. Mandibles from the Community-Based Wildlife Health Surveillance program (2014-2021) listed by sex and harvest location (closest community or geographic landmark).

Location Of Harvest	***Female***	***Male***	***Unknown***	***Total***
Cambridge Bay	14	31	5	50
Ulukhaktok	44	29	3	76
Lady Franklin Point	7	3		10
Kent Peninsula	2	23		25
Kugluktuk	58	31	7	96
Unknown			3	3*
Total	**125**	**117**	**18**	**260**

*Three muskoxen were not assigned a specific harvest location but were from Victoria Island.
