# Supplementary figures and images for: A refined guide for aging muskoxen (Ovibos moschatus) based on mandibular examination
Source: PLoS One. 2025 Sep 24;20(9):e0328994. doi: 10.1371/journal.pone.0328994 (PMC12459791; doi:10.1371/journal.pone.0328994)

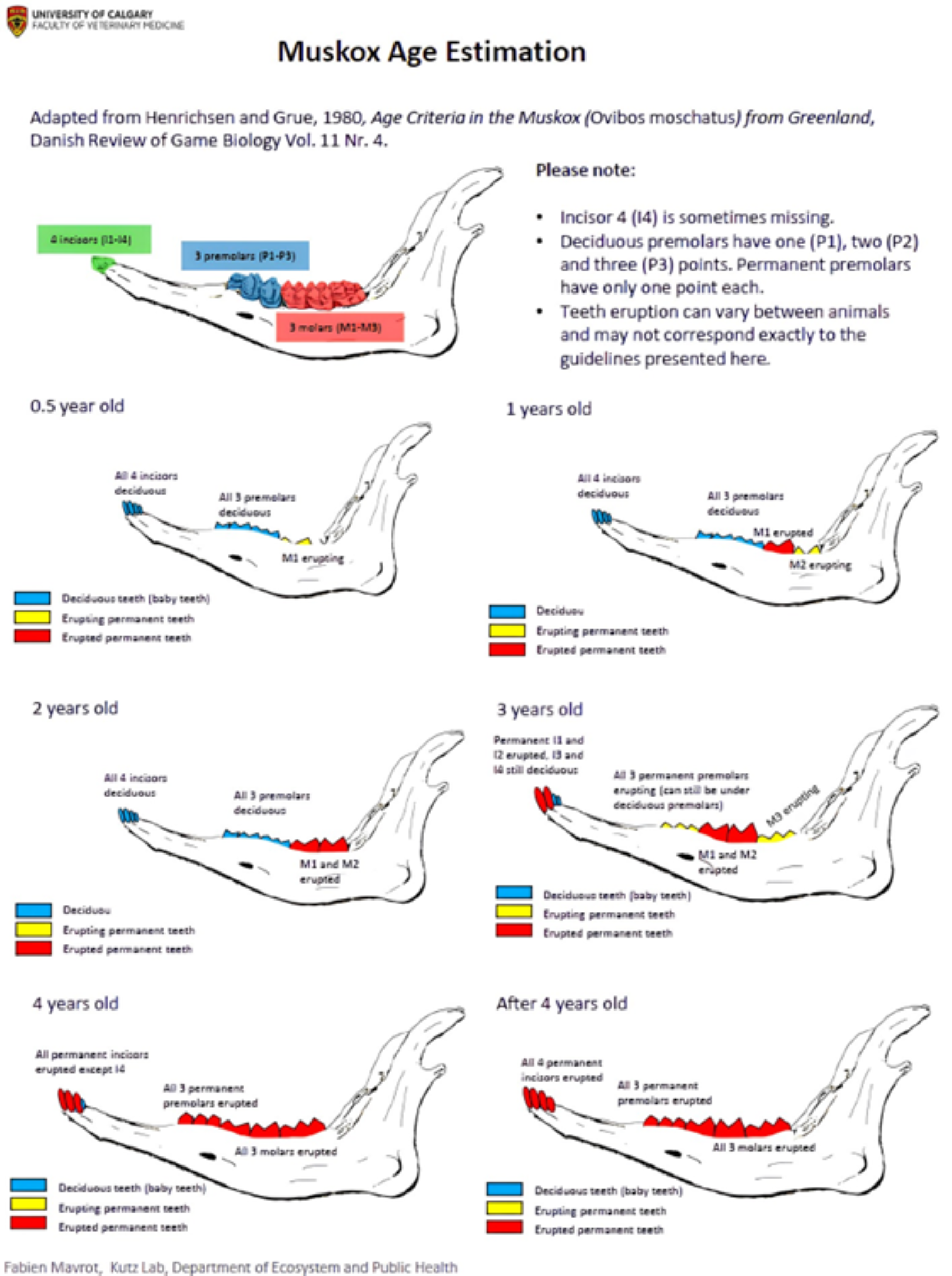

Supplement: S2 Fig — Adapted from Henrichsen and Grue (1980). (TIF) [file pone.0328994.s002.tif]

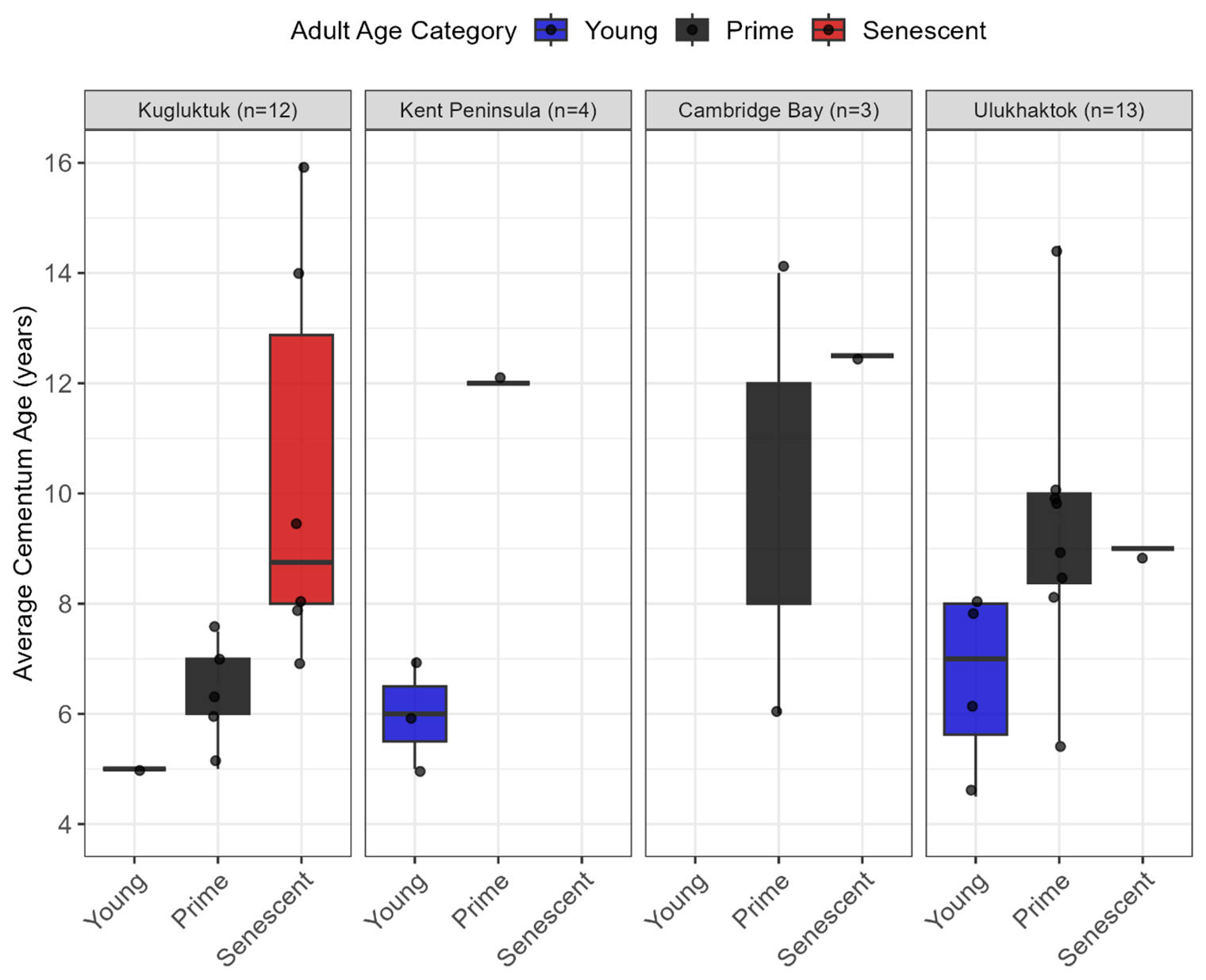

Supplement: S8 Fig — Estimated ages of 32 muskoxen from the Community-based Wildlife Health Surveillance program determined through cementum annuli analysis, shown by harvest community and assigned age category. Age categories were assigned based on visual assessment of tooth wear, with greater wear indicating older age classifications. (TIF) [file pone.0328994.s008.tif]
