# Supplementary material for: A refined guide for aging muskoxen (Ovibos moschatus) based on mandibular examination
Source: PLoS One. 2025 Sep 24;20(9):e0328994. doi: 10.1371/journal.pone.0328994 (PMC12459791; doi:10.1371/journal.pone.0328994)
Supplement: S3 Table — Radiographs and photographs of muskox incisor arcades and molars, showing known months of death and estimated age in years and months until all permanent teeth complete eruption. Primary incisors and molars were denoted with a lowercase “i” and “p” and permanent incisors and premolars were denoted with capital “I” and “P” respectively. Permanent molars are labeled with a capital “M”. Eruption stage 1–starting to erupt through the bone; stage 2—continuing to erupt through the bone; stage 3—complete eruption through both bone into oral cavity. Incisors that are missing either suffered pre-mortem fractures or were extracted for cementum annuli analysis. (PDF) [file pone.0328994.s003.pdf]

S3 Table. Radiographs and photographs of muskox incisor arcades and molars, showing known months of death and estimated age in years and months until all permanent teeth complete eruption. Primary incisors and molars were denoted with a lowercase “i” and “p” and permanent incisors and premolars were denoted with capital “I” and “P” respectively. Permanent molars are labeled with a capital “M”. Eruption stage 1- starting to erupt through the bone; stage 2—continuing to erupt through the bone; stage 3—complete eruption through both bone into oral cavity. Incisors that are missing either suffered pre-mortem fractures or were extracted for cementum annuli analysis.

<table>
<tr><th>Season of Death</th><th>Age</th><th>Radiographs</th><th>Photographs</th><th>Description</th></tr>
<tr><td rowspan="2">Spring (Apr-Jun)</td><td rowspan="2">calf
1-2 mo
(n= 1)</td><td>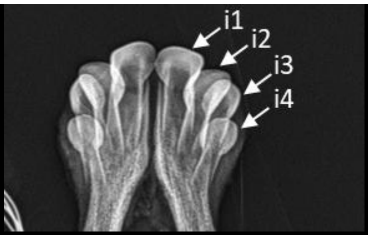
</td><td>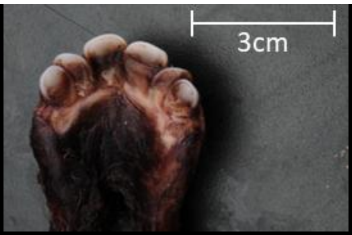
</td><td>i_1, i_2, and i_3 stage 2, i_4 unerupted*</td></tr>
<tr><td>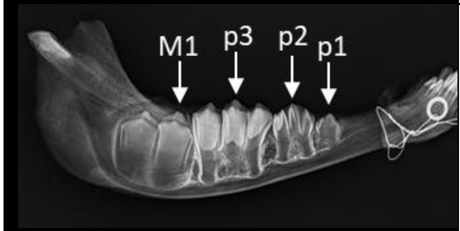
</td><td>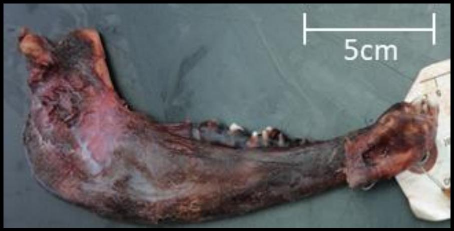
</td><td>p_1 stage 1, p_2 and p_3 stage 2</td></tr>
<tr><td rowspan="2">Autumn (Oct-Dec)</td><td rowspan="2">calf
6-8 mo
(n= 5)</td><td>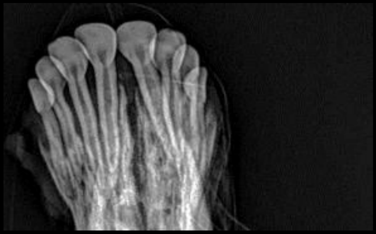</td><td>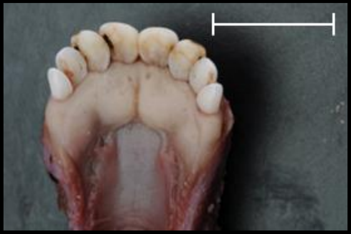</td><td>All primary incisors erupted</td></tr>
<tr><td>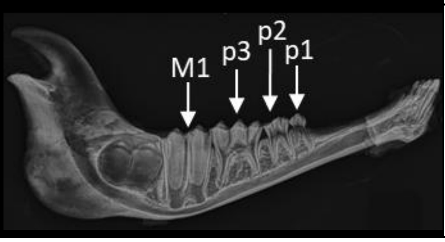
</td><td>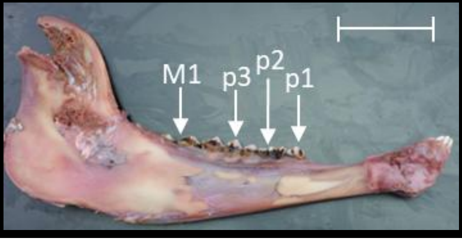
</td><td>M_1 stage 2, rostral cusp visible in oral cavity. M_2 developing</td></tr>
</table>

Winter (Jan-Mar)	calf 9-11 mo (n= 10)	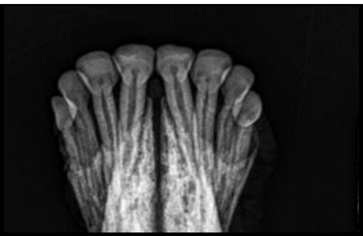	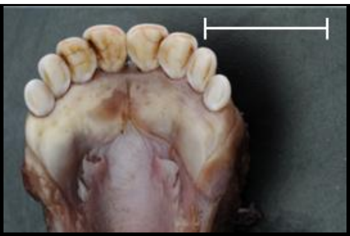	No permanent incisors developing
		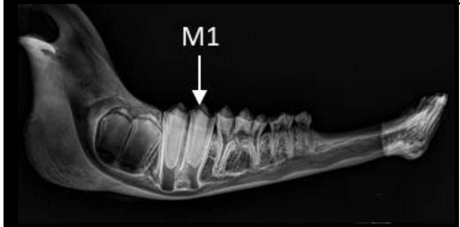	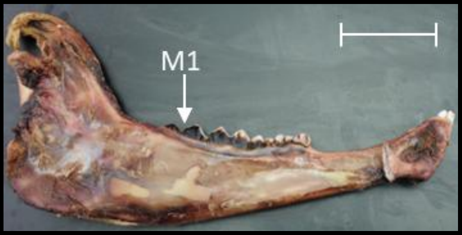	M_1 near stage 3. Caudal cusp visible in oral cavity. M_2 developing
Spring (Apr-Jun)	1 yo 12-14 mo (n= 10)	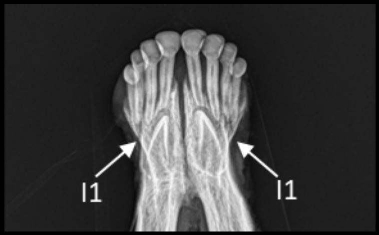	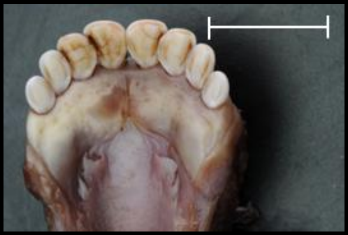	I_1 developing. Only primary incisors erupted
		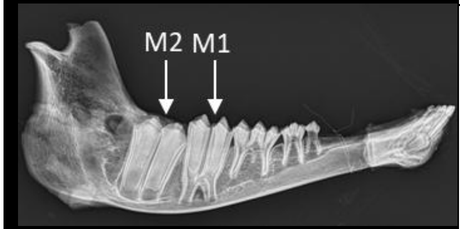	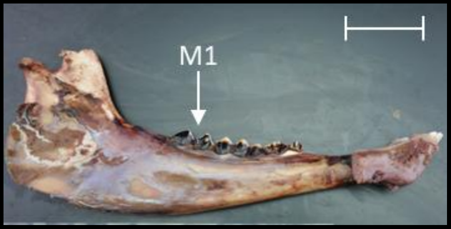	M_1 stage 3 & M_2 stage 1
Summer (Jul-Sep)	1 yo 15-17 mo (n= 2)	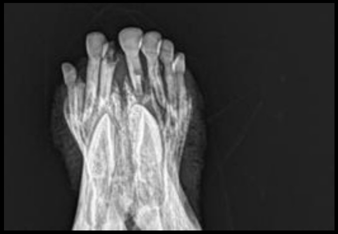	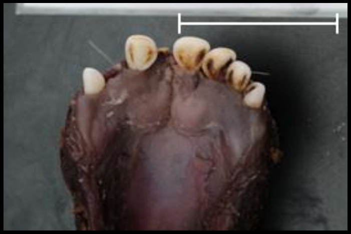	I_1 developing
		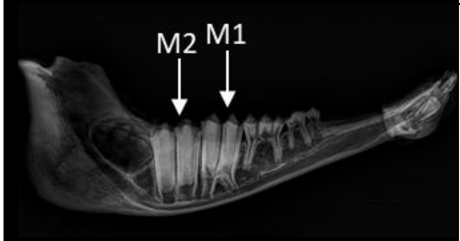	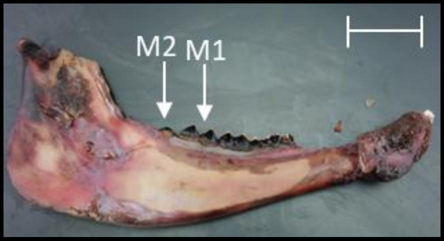	M_2 stage 2. Rostral cusp visible in oral cavity.
Autumn (Oct-Dec)	1 yo 18-20 mo (n= 6)	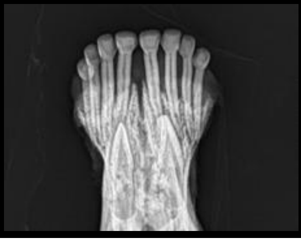	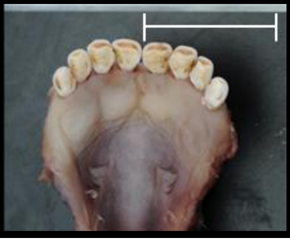	I_1 developing

<table>
<tr><td></td><td></td><td>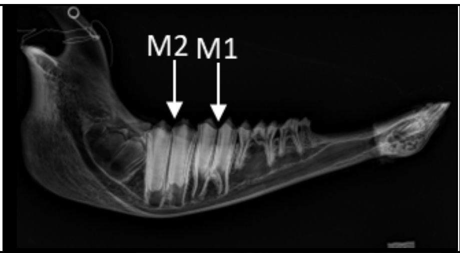</td><td>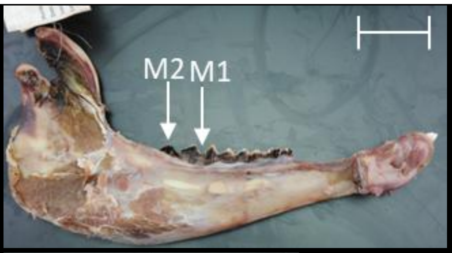</td><td>M_2 stage 2 with minimal change.</td></tr>
<tr><td rowspan="2">Winter (Jan-Mar)</td><td rowspan="2">1 yo 21-23 mo (n= 14)</td><td>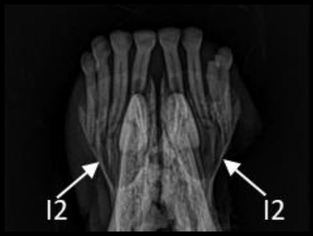</td><td>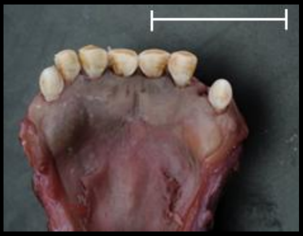</td><td>I_1 mineralized below gingiva. I_2 beginning to develop</td></tr>
<tr><td>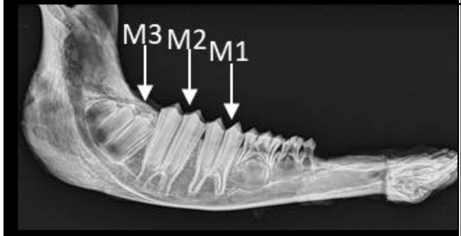</td><td>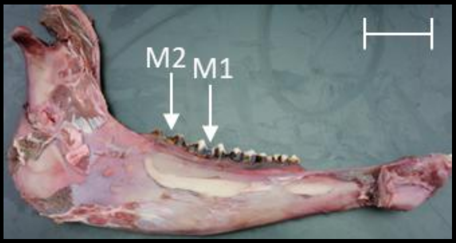</td><td>M_2 near stage 3. Caudal cusp visible in oral cavity. M_3 developing</td></tr>
<tr><td rowspan="2">Spring (Apr-Jun)</td><td rowspan="2">2 yo 24-26 mo (n= 9)</td><td>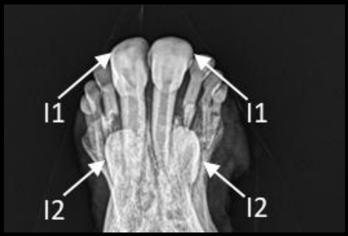</td><td>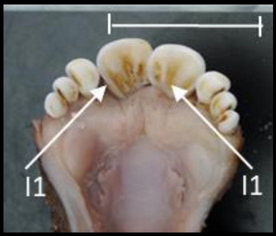</td><td>I_1 erupted. I_2 mineralized below gingiva</td></tr>
<tr><td>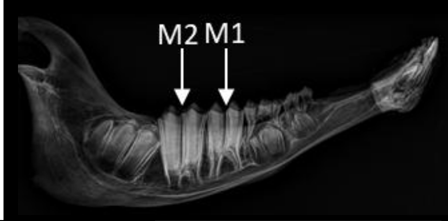</td><td>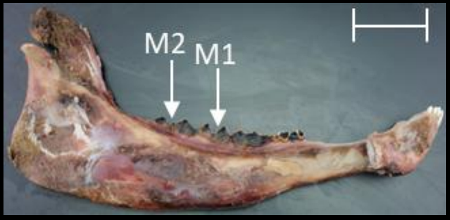</td><td>M_2 stage 3. P_3 & P_2 developing</td></tr>
<tr><td rowspan="2">Autumn (Oct-Dec)</td><td rowspan="2">2 yo 30-32 mo (n= 3)</td><td>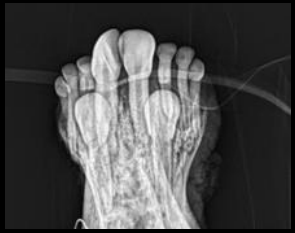</td><td>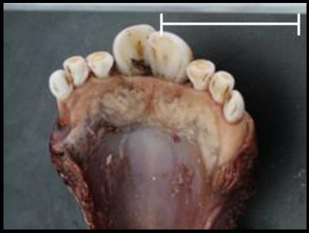</td><td>I_1 erupted. I_2 below gingiva</td></tr>
<tr><td>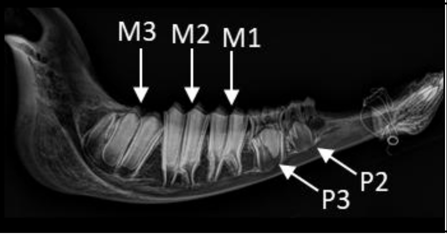</td><td>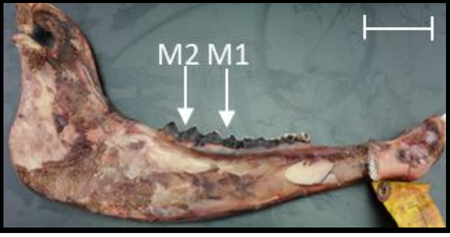</td><td>M_3 stage 1. P_2 & P_3 stage 1</td></tr>
</table>

Winter (Jan-Mar)	2 yo 33-35 mo (n= 10)	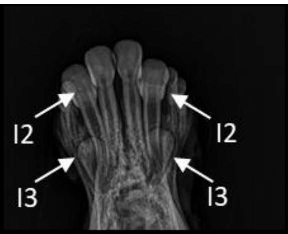	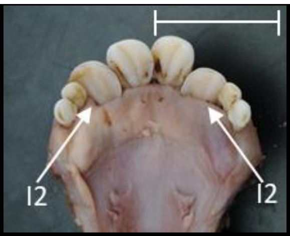	I_2 near stage 3 & I_3 developing
		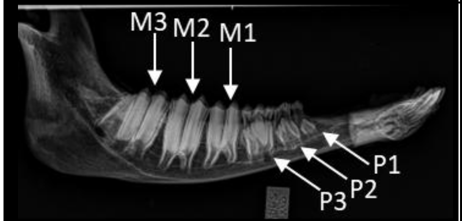	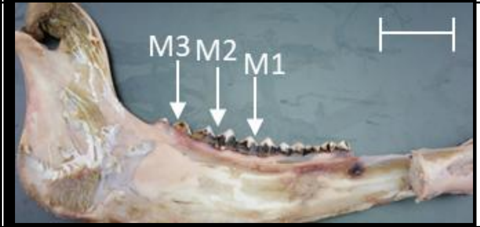	M_3 stage 2. Cusp 1 & tip of cusp 2 visible. P_1, P_2, & P_3 stage 1
Spring (Apr-Jun)	3 yo 36-38 mo (n= 2)	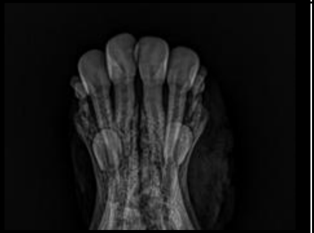	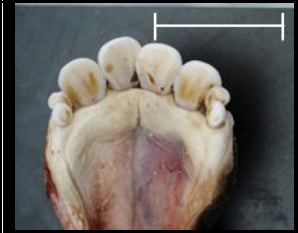	I_1 & I_2 erupted. I_3 mineralized
		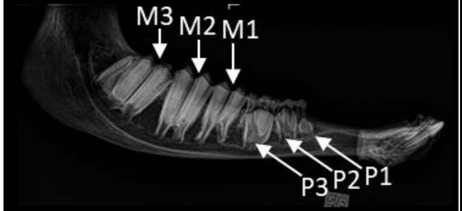	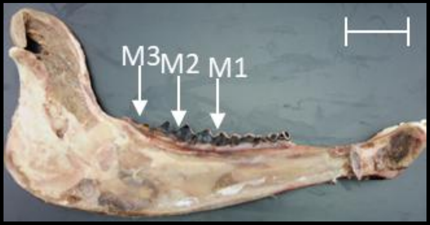	M_3 stage 2. P_1, P_2, & P_3 stage 1. Not much change
Autumn (Oct-Dec)	3 yo 42-44 mo (n= 2)	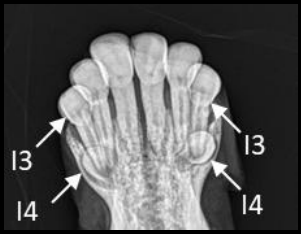	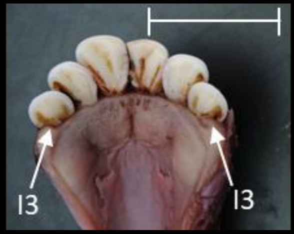	I_3 erupted. I_4 developing. No primary I_4s present
		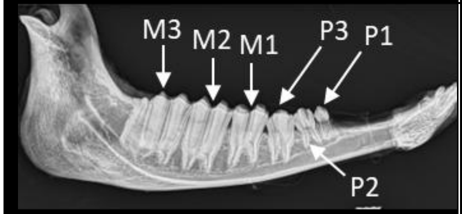	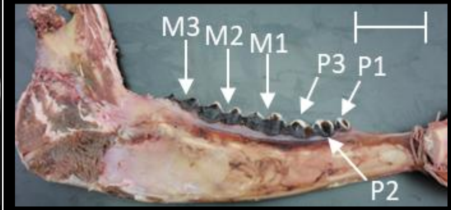	M_3 stage 2. P_1, P_2, & P_3 near stage 3
Winter (Jan-Mar)	3 yo 45-47 mo (n= 7)	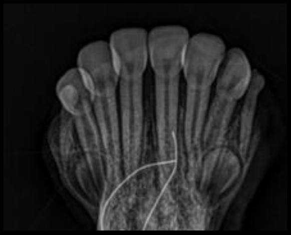	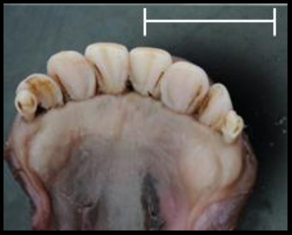	I_1, I_2, & I_3 erupted. I_4 developing
		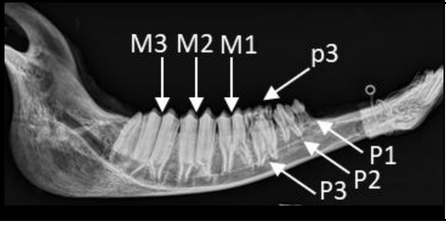	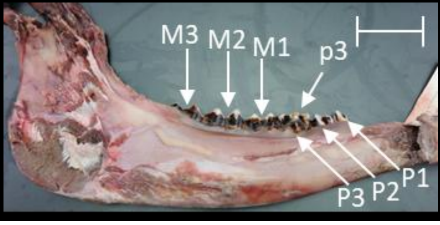	M_3 stage 2. P_1 & P_2 stage 3, P_3 near stage 3

<table>
<tr><td rowspan="2">Autumn (Oct-Dec)</td><td rowspan="2">4 yo
54-56
mo
(n= 4)</td><td>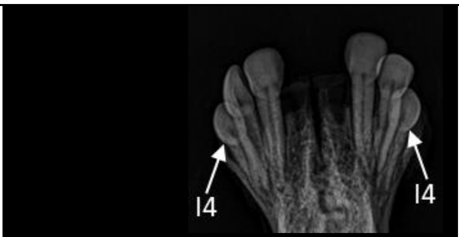
</td><td>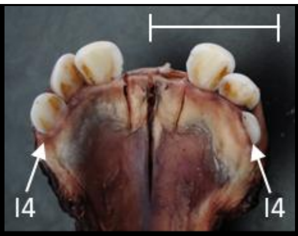
</td><td>I_4 stage 2</td></tr>
<tr><td>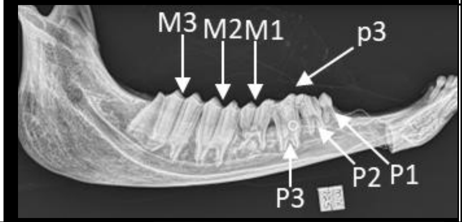
</td><td>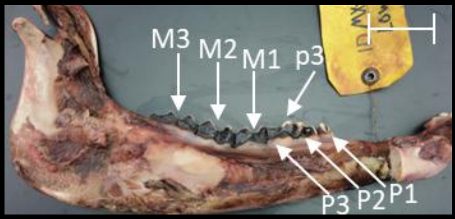
</td><td>M_3 near stage 3. P_3 stage 3 with primary precursor</td></tr>
<tr><td rowspan="2">Winter (Jan-Apr)</td><td rowspan="2">4 yo
57-60
mo
(n= 12)</td><td>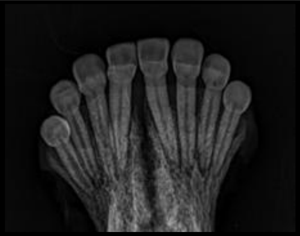</td><td>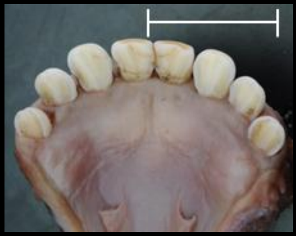</td><td>I_1, I_2, I_3, I_4 erupted</td></tr>
<tr><td>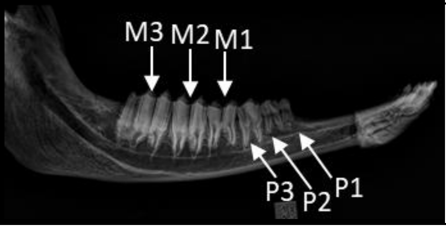
</td><td>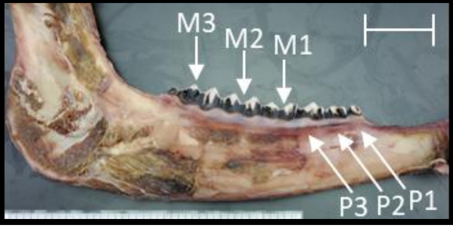
</td><td>M_3 stage 3</td></tr>
</table>
