# Supplementary material for: A refined guide for aging muskoxen (Ovibos moschatus) based on mandibular examination
Source: PLoS One. 2025 Sep 24;20(9):e0328994. doi: 10.1371/journal.pone.0328994 (PMC12459791; doi:10.1371/journal.pone.0328994)
Supplement: S4 Table — Stages of tooth eruption were assessed for each tooth, based on visual inspection of the mandibles. The stages are defined as follows: NE (No Eruption–white): tooth not erupted into the oral cavity; IE (Initial Eruption–lightest grey): first appearance of the tooth through the gingiva; PE (Partial Eruption–light grey): half of the tooth visible in the oral cavity; NCE (Near Complete Eruption–medium grey): tooth almost fully erupted through the gingiva; CE (Complete Eruption–dark grey): tooth completely emerged in its functional position in oral cavity. Primary incisors and molars were denoted with a lowercase “i” and “p” and permanent incisors and premolars were denoted with capital “I” and “P” respectively. Permanent molars are labeled with a capital “M”. (PDF) [file pone.0328994.s004.pdf]

S4 Table. Tooth eruption patterns from gross examination of muskox mandibles by age (in months) until complete eruption of all teeth. Stages of tooth eruption were assessed for each tooth, based on visual inspection of the mandibles. The stages are defined as follows: NE (No Eruption- white): tooth not erupted into the oral cavity; IE (Initial Eruption- lightest grey): first appearance of the tooth through the gingiva; PE (Partial Eruption- light grey): half of the tooth visible in the oral cavity; NCE (Near Complete Eruption- medium grey): tooth almost fully erupted through the gingiva; CE (Complete Eruption- dark grey): tooth completely emerged in its functional position in oral cavity. Primary incisors and molars (grouped under "Premolars") were denoted with a lowercase "i" and "p" and permanent incisors and premolars were denoted with capital "I" and "P" respectively. Permanent molars are labeled with a capital "M".

Season of Death	Age in Months	Incisors				Premolars			Molars		
		I_1	I_2	I_3	I_4	P_1	P_2	P_3	M_1	M_2	M_3
Spring (Apr-Jun)	1-2	i_1 NCE	i_2 NCE	i_3 NCE	i_4 NE	p_1 IE	p_2 PE	p_3 PE	M_1 NE	M_2 NE	M_3 NE
Summer (Jul-Sep)	3-5	i_1 CE	i_2 CE	i_3 CE	i_4 CE	p_1 CE	p_2 CE	p_3 CE	M_1 IE	M_2 NE	M_3 NE
Autumn (Oct-Dec)	6-8	I_1 NE	I_2 NE	I_3 NE	I_4 NE	P_1 NE	P_2 NE	P_3 NE	M_1 PE	M_2 NE	M_3 NE
Winter (Jan-Mar)	9-11	I_1 NE	I_2 NE	I_3 NE	I_4 NE	P_1 NE	P_2 NE	P_3 NE	M_1 NCE	M_2 NE	M_3 NE

Spring (Apr-Jun)	12-14	I_1 NE	I_2 NE	I_3 NE	I_4 NE	P_1 NE	P_2 NE	P_3 NE	M_1 CE	M_2 NE	M_3 NE
Summer (Jul-Sep)	15-17	I_1 NE	I_2 NE	I_3 NE	I_4 NE	P_1 NE	P_2 NE	P_3 NE	M_1 CE	M_2 IE	M_3 NE
Autumn (Oct-Dec)	18-20	I_1 NE	I_2 NE	I_3 NE	I_4 NE	P_1 NE	P_2 NE	P_3 NE	M_1 CE	M_2 PE	M_3 NE
Winter (Jan-Mar)	21-23	I_1 NE	I_2 NE	I_3 NE	I_4 NE	P_1 NE	P_2 NE	P_3 NE	M_1 CE	M_2 NCE	M_3 NE
Spring (Apr-Jun)	24-26	I_1 CE	I_2 NE	I_3 NE	I_4 NE	P_1 NE	P_2 NE	P_3NE*	M_1CE	M_2 CE	M_3 NE
Autumn (Oct-Dec)	30-32	I_1 CE	I_2 NE	I_3 NE	I_4 NE	P_1 NE	P_2 NE	P_3 NE	M_1 CE	M_2 CE	M_3 IE
Winter (Jan-Mar)	33-35	I_1 CE	I_2 CE	I_3 NE	I_4 NE	P_1 NE	P_2 NE	P_3 NE	M_1 CE	M_2 CE	M_3 PE rostral cusp visible
Spring (Apr-Jun)	36-38	I_1 CE	I_2 CE	I_3 NE	I_4 NE	P_1 NE	P_2 NE	P_3 NE	M_1 CE	M_2 CE	M_3 PE rostral cusp visible
Autumn (Oct-Dec)	42-44	I_1 CE	I_2 CE	I_3 CE	I_4 NE	P_1 NCE	P_2 NCE	P_3 NCE	M_1 CE	M_2 CE	M_3 PE rostral and middle cusp visible

Winter (Jan-Mar)	45-47	I_1 CE	I_2 CE	I_3 CE	I_4 NE	P_1 CE	P_2 NCE	P_3 NCE	M_1 CE	M_2 CE	M_3 PE rostral and middle cusp visible
Spring (Apr-Jun)	48-50	I_1 CE	I_2 CE	I_3 CE	I_4 CE*	P_1 CE	P_2 CE	P_3 NCE	M_1 CE	M_2 CE	M_3 PE rostral and middle cusp visible
Autumn (Oct-Dec)	54-56	I_1 CE	I_2 CE	I_3 CE	I_4 CE	P_1 CE	P_2 CE	P_3 CE*	M_1 CE	M_2CE	M_3 NCE caudal cusp visible
Winter (Jan-Apr)	57-60	I_1 CE	I_2 CE	I_3 CE	I_4 CE	P_1 CE	P_2 CE	P_3 CE	M_1 CE	M_2 CE	M_3 CE

*Primary precursor may still be present concealing the permanent premolar.
