# Supplementary material for: A refined guide for aging muskoxen (Ovibos moschatus) based on mandibular examination
Source: PLoS One. 2025 Sep 24;20(9):e0328994. doi: 10.1371/journal.pone.0328994 (PMC12459791; doi:10.1371/journal.pone.0328994)
Supplement: S5 Table — The stages are defined as follows: NE (No Eruption–white): Tooth not yet erupted into the oral cavity; PE (Partial Eruption–light grey): Less than half of the tooth visible in the oral cavity; NCE (Near Complete Eruption–medium grey): Tooth nearly emerged through the gingiva; CE (Complete Eruption–dark grey): Tooth completely emerged and in its functional position. Primary incisors and molars were denoted with a lowercase “i” and “p” and permanent incisors and premolars were denoted with capital “I” and “P” respectively. Permanent molars are labeled with a capital “M”. (PDF) [file pone.0328994.s005.pdf]

S5 Table. Tooth eruption patterns for muskoxen across ages: calf, 1 yo, 2 yo, 3 yo, 4 yo, and 5 yo^{+}. The stages are defined as follows: NE (No Eruption - white): Tooth not yet erupted into the oral cavity; PE (Partial Eruption - light grey): Less than half of the tooth visible in the oral cavity; NCE (Near Complete Eruption - medium grey): Tooth nearly emerged through the gingiva; CE (Complete Eruption - dark grey): Tooth completely emerged and in its functional position. Primary incisors and molars (grouped under "Premolars") were denoted with a lowercase "i" and "p" and permanent incisors and premolars were denoted with capital "I" and "P" respectively. Permanent molars are labeled with a capital "M".

	**Incisors**				**Premolars**			**Molars**		
**Age**	**I_1**	**I_2**	**I_3**	**I_4**	**P_1**	**P_2**	**P_3**	**M_1**	**M_2**	**M_3**
Calf	i_1 CE	i_2 CE	i_3 CE	i_4 CE	p_1 CE	p_2 CE	p_3 CE	M_1 PE	M_2 NE	M_3 NE
1 yo	I_1 NE	I_2 NE	I_3 NE	I_4 NE	P_1 NE	P_2 NE	P_3 NE	M_1 CE	M_2 PE	M_3 NE
2 yo	I_1 CE	I_2 NE	I_3 NE	I_4 NE	P_1 NE	P_2 NE	P_3 NE	M_1 CE	M_2 CE	M_3 PE
3 yo	I_1 CE	I_2 CE	I_3 NE	I_4 NE	P_1 PE	P_2 PE	P_3 PE	M_1 CE	M_2 CE	M_3 PE
4 yo	I_1 CE	I_2 CE	I_3 CE	I_4 NE*	P_1 CE	P_2 CE	P_3 CE	M_1 CE	M_2 CE	M_3 NCE
5 yo^{+}	I_1 CE	I_2 CE	I_3 CE	I_4 CE	P_1 CE	P_2 CE	P_3 CE	M_1 CE	M_2 CE	M_3 CE
