# Supplementary material for: A refined guide for aging muskoxen (Ovibos moschatus) based on mandibular examination
Source: PLoS One. 2025 Sep 24;20(9):e0328994. doi: 10.1371/journal.pone.0328994 (PMC12459791; doi:10.1371/journal.pone.0328994)
Supplement: S6 Table — Estimates for two sections from the left I1 (if available) of muskoxen of known age from captive facilities. The grade indicates the confidence in the age estimate, where A–high confidence, B–some uncertainty, C–more uncertainty. Notes include information on abnormal histology, cementum damage. (PDF) [file pone.0328994.s006.pdf]

S6 Table. Cementum annuli analysis results for two sections from the left I_1 (if available) of muskoxen of known age from captive facilities. The grade indicates the confidence in the age estimate, where A-high confidence, B-some uncertainty, C- more uncertainty. Notes include information on abnormal histology and cementum damage.

**Animal ID**	**Location**	**Sex**	**Known Age**	**Cem Age Slice 1**	**Cem Age Slice 2**	**Grade**	**Notes**
Leo	AK	Male	**19**	14	15	A	
Elim	AK	Male	**14**	9	10	A	
GeorgeAnn	AK	Female	**27**	8	10	A	
MOX 1166	AK	Female	**11**	5	8	A	
MOX 347	AK	Female	**9**	4	5	B	
MOX 619	AK	Male	**6**	5	5	A	
Makuktok	SK		**8**	7	7	B	
Bruiser	SK	Male	**9**	6	6	B	Abnormal Histology (AH)
Pappy	SK	UKN	**5**	3	3	A	
Esav	SK	UKN	**8**	6	6	A	Cementum Damage
What	SK	UKN	**5**	5	5	A	
Amos	SK	Male	**6**	4	5	A	
Sonny	AB	Male	**15**	10	9	A	AH
22166	MB	Male	**6**	4	5	B	
Tanner	MB	Male	**6**	5	5	A	
