# Supplementary material for: A refined guide for aging muskoxen (Ovibos moschatus) based on mandibular examination
Source: PLoS One. 2025 Sep 24;20(9):e0328994. doi: 10.1371/journal.pone.0328994 (PMC12459791; doi:10.1371/journal.pone.0328994)
Supplement: S7 Table — Ratio between mean incisor arcade width and total mandible length for adult muskoxen (5 years or older) from Victoria Island and the adjacent mainland. T-tests were done to compare ratio by sex. (PDF) [file pone.0328994.s007.pdf]

S7 Table. Mean incisor arcade width to total mandible length ratio for adult muskoxen (5 years or older) from Victoria Island and the adjacent mainland. Independent t-tests were conducted to compare the ratios by sex. Confidence intervals (95% CI) and t-test statistics are reported for each comparison.

Sex	Mean Ratio Victoria Island	Mean Ratio Adjacent Mainland	t-value	df	p-value	95% CI
Males	0.0841	0.0937	-4.79	53	<0.0001	[-0.0135, -0.0055]
Females	0.0877	0.0957	-5.28	56.5	<0.0001	[-0.0111, -0.0050]
